# Tracheal and bronchial involvement in colitis ulcerosa – a colo-bronchitic syndrome? A case report and some additional considerations

**DOI:** 10.3205/000207

**Published:** 2015-03-30

**Authors:** Peter von Wichert, Peter Barth, Goetz von Wichert

**Affiliations:** 1 Department of Medicine, Philipps-University of Marburg, Germany; 2 Department of Pathology, Philipps-University of Marburg, Germany; 3 Department of Medicine, University of Ulm, Germany

**Keywords:** inflammatory bowel disease, ulcerative colitis, Crohn’s disease, bronchitis, bronchiectasis

## Abstract

Systemic involvement is well known in patients with inflammatory bowel diseases (IBD), but there are only few data looking to Crohn’s disease (CD) and ulcerative colitis (UC) separately instead of lumping together both entities to IBD. The frequency of bronchial involvement in UC is not yet exactly analysed but reported to be rare. We asked 100 patients with UC for bronchial complaints, and found in 13 patients a bronchial affection. From reports in the literature it is known that sometimes a bronchial involvement in patients with UC can affect the whole bronchial tree including small bronchi. The involvement of bronchial system in UC is obviously more prominent than previously thought and may fulfil the criteria for a separate syndrome. These relations may have consequences for pathogenetic understanding of UC as well as bronchitis and also consequences for treatment regimes.

## Zusammenfassung

Beteiligungen systemischer Organe sind bei entzündlichen Darmerkrankungen bekannt, bisher wurden aber Morbus Crohn und Colitis kaum separat bezüglich ihrer Systembeteiligung betrachtet. So ist die Häufigkeit bronchialer Beteiligung speziell bei Colitis ulcerosa unbekannt, wird in der Literatur als selten angesehen. Wir fragten 100 Patienten mit Colitis ulcerosa nach bronchialen Beschwerden und fanden bei 13 Patienten eine floride Bronchitis. Die bronchiale Beteiligung ist offensichtlich häufiger als bisher angenommen und könnte die Kriterien eines Syndroms erfüllen. Für pathogenetisches Verständnis und therapeutische Konsequenzen sowohl von Colitis wie Bronchitis würde das bedeutsam sein.

## Introduction

While serving a patient with severe necrotising bronchitis who additionally developed an ulcerative colitis we suspected common features of both epithelial inflammations. The systemic character of inflammatory bowel diseases (IBD) is underlined by extra-intestinal manifestations in cases of Crohn’s disease (CD) as well as ulcerative colitis (UC). Affections of joints, eyes, and skin are reported, less frequently of the lungs and the liver and other organs, together in nearly 25% of patients with IBD [1], [2], [3], [4], [5]. A very thorough description of lung involvement in IBD was given by Camus et al. [2], but in this paper and in the majority of reports no differentiation was made between CD and UC concerning systemic involvement. To this day the common manifestations of UC and bronchial involvement are mostly seen as accidental. In our paper we put forward the idea that there may be a more causal relationship between both conditions in form of a colo-bronchitic syndrome.

## Methods

### Case description 

**Figure 1:**
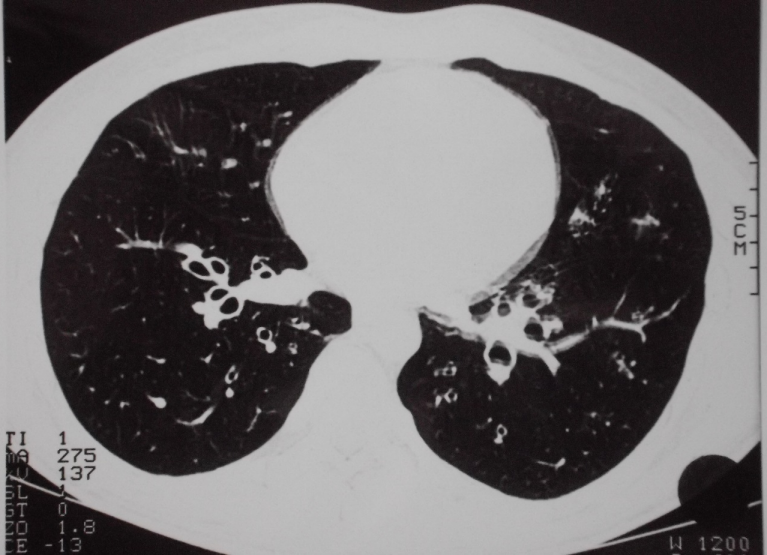
Thorax CT shows cylindrical bronchiectasis and thick bronchial walls. Lung parenchyma is mostly unchanged. (Department of Radiology of Philipps-University Marburg)

**Figure 2:**
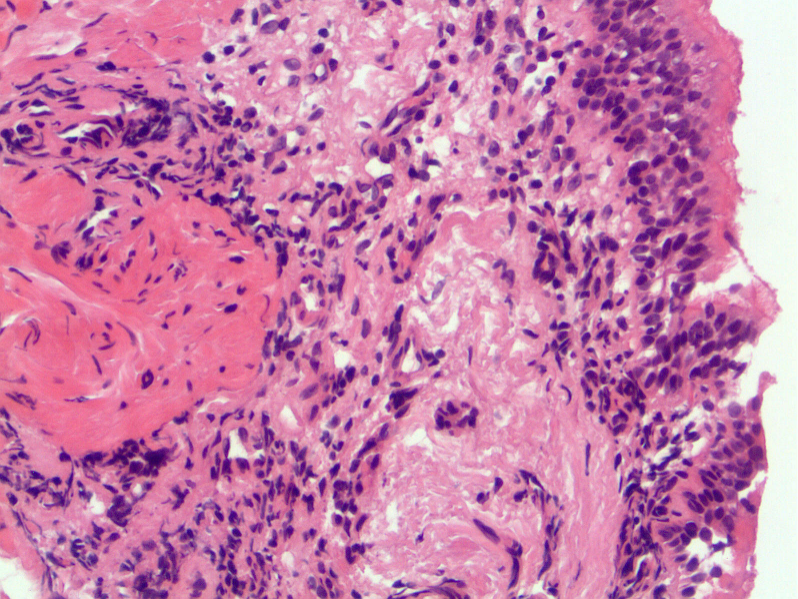
Bronchial mucosa shows a patchy interstitial lymphocytic infiltrate. The epithelium is hyperplastic and discloses few intraepithelial lymphocytes.

**Figure 3:**
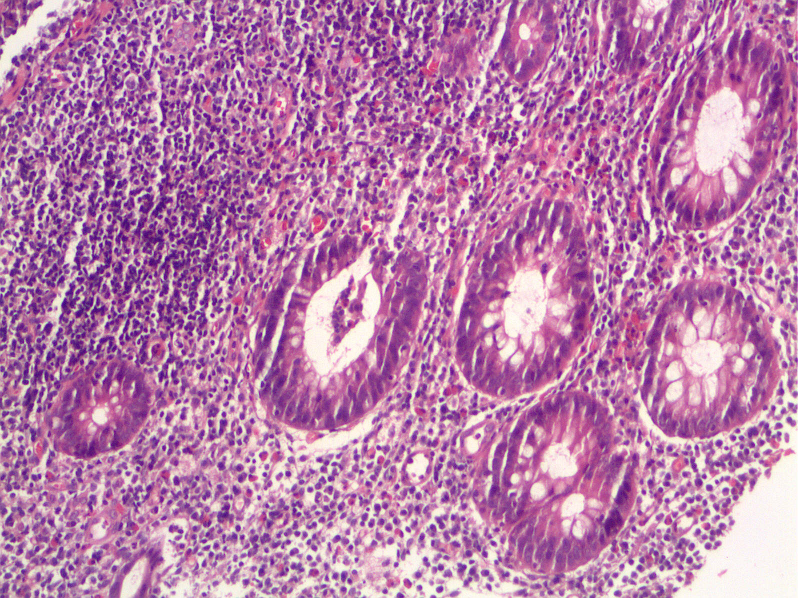
Colonic mucosa biopsies reveal a diffuse interstitial infiltrate mainly composed of lymphocytes with admixed plasma cells and eosinophils, few intraepithelial granulocytes and crypt abscesses. There is slight mucosal architectural distortion.

A non-smoking 22-year-old male patient reported strong bronchitic complaints, permanent cough, massive purulent expectoration and dyspnoea during exercise for two years before visiting the hospital. He had infrequently bloody stool, but unfortunately this symptom was overlooked at first consultation. Lung function was borderline normal, but pO_2_ was only 75.5 mmHg. There were no signs of immunologic disorders or allergy. HR-CT presented thick bronchial walls and tubular bronchiectasis (Figure 1 (Fig. 1)). Bronchoscopically a massive suppurative bronchitis with mucoid impaction was seen, but bacteriological and virus findings were negative. Indication of cystic fibrosis or bronchopulmonary aspergillosis was not seen. Histology is shown in Figure 2 (Fig. 2). The patient was successfully treated with antibiotics and steroids and he became slowly but continously better. A bronchoscopic control half a year later showed a macroscopically and histologically unchanged situation. At that time he developed abdominal complaints with bloody diarrhoea. Colonoscopy showed macroscopically UC, histologically confirmed (Figure 3 (Fig. 3)). The treatment led to clinical improvement of the abdominal and bronchial complaints. Nevertheless 15 months later bronchitis and colitis were still present if analysed endoscopically although clinically silent. 

### Pilot study on relation between bronchial complaints and UC

To understand the frequency of the affection of the bronchial tree in patients with UC we have done a pilot study in an outpatient setting to evaluate relations between intestinal and bronchial involvement. 

100 consecutive patients with histologically proven UC visiting the IBD outpatient service of the University Hospital of Ulm/Germany were asked if they ever had symptomatic bronchitic complaints, cough and sputum, and if yes, when and in which relation to UC this has happened. Smoking was judged positive if the patient actually smoke. In this pilot study we didn’t ask for severity or stage of the disease nor which treatments have been used. Table 1 (Tab. 1) shows the results. 13 patients out of hundred have reported to have bronchial complaints, 10 of them were smokers – 77%.

We formed 3 groups according to the duration of UC. The proportion of bronchial involvement was greater in those patients with long lasting UC. Smokers were found in all groups with and without bronchitic complaints. The proportion of bronchial symptoms in relation to UC was more evident in the patients with a shorter duration of UC. All 3 patients in the group III reported an exacerbation of the bronchial impairment simultaneously with an exacerbation of UC. The patient in group III who was non-smoker was the one presented above with a most severe tracheobronchitis.

## Discussion

Clinical observations may help to find biological relations which may induce more detailed investigations to get new pathogenetic insights. Clinical observations stimulate pathogenetic reflections which may otherwise perish in the statistical mass.

### The lungs in UC

The affection of the lungs in cases of IBD was first reported by [6], [7], [8] and later on in detail by [9], [10]. The mechanisms of the pulmonary affection in IBD are not clear, immunologic processes are likely but not clearly elaborated [2], [3], [8]. The literature describes different reactions of pulmonary tissue. Interstitial affections not only related to the mesalazin therapy [11], [12], [13], [14], and granulomatous and vasculitic changes are reported. Studies show that interstitial affections are more frequent in patients with MC than in those with UC [2], [10]. A growing body of evidence describes isolated affections of the bronchial tree in UC [2], [8], [15]. In most reports both IBDs are taken together, particularly in relation to the systemic affections, although IBDs are different in pathogenesis, morphology and in genetic data [3], [16]. Additionally many papers analysing the prevalence of extraintestinal manifestations of IBD are not dealing with lung involvement in UC [3], [17], [18] although a bronchial involvement was reported early in X-ray studies [19], but rated as seldom [20]. Only a report of Herrlinger et al. [21] gives some data on the frequency of lung involvement in cases of IBD. They show in 39% of CD-patients and in 45% of UC-patients at least one pathological lung function test pointing to an at least subclinical pulmonary involvement in IBD. According to the published case reports the affection of the bronchial system is prominent in UC in contrast to CD. It is not clear if this may have a connection to smoking habit [3], [22] or to immunological or microbiological processes, or a general dysfunction of the epithelial barrier [3] or the common embryonic ancestry. There are no studies done to analyse these clinical observations concerning a pathogenetic background. 

The 13% of UC-patients with bronchial complaints in our series were picked up only asking for clinically apparent symptoms. We may have overlooked persons with subclinical forms of bronchitis. Although 10 of the 13 were smokers it is unknown if smoking alone was responsible for the bronchitic complaints, or if it acts as an additional noxious event to a basic pathological process. Interestingly also in the group without bronchial complaints smoking was frequent.

Table 2 (Tab. 2) collects published cases of UC with lung problems. Looking at these reports it appears likely that a common occurrence of UC and bronchitis could be a result of a compound syndrome, which should then be denoted as a “colo-bronchitic” syndrome. Lung affections in cases of IBD are reported as being seldom [20]. The reason may be that the described coherence is overlooked because oligosymptomatic respiratory complaints are considered often as unimportant or easily related to smoking, which is frequent in patients suffering from UC [3], [22]. The proportion of smokers in our group of patients with bronchial involvement is high (77%), in contrast to Camus et al. [2] who found bronchial involvement mostly in nonsmokers. In the whole series of our patients the smoking rate of 32% is even higher than the average rate of smoking in Germany, which is actually 25%. Patients with UC are mostly cared for by nonpulmonary physicians and therefore a more detailed analysis of pulmonary involvement probably is not undertaken. The oligosymptomatic or even asymptomatic course of bronchial involvement in many patients makes it difficult to pick up those patients early [15]. However looking carefully at these patients, it becomes clear that pulmonary symptoms are relatively frequent [21], although in that study the tests are mainly aimed at lung parenchyma rather than the bronchial system. In the series of Herrlinger [21] more than 50% of the patients have CD rather than UC, just as in a series of Camus et al. [2] who likewise do not differentiate between patients with UC or CD or between functional changes concerning lung parenchyma or the bronchi. Our results show that a differentiation between CD and UC is imperative to recognize systemic effects. Reports in the literature relating to the affection of the lungs in IBD suggest that in UC the bronchial involvement is a prominent symptom (Table 1 (Tab. 1)) [12], [23], although there are also reports on parenchymal involvement in UC [23]. A population study in Sweden has shown that in patients with chronic obstructive pulmonary disease (COPD) the frequency of IBD and particularly UC is increased beyond the frequency reported for smoking alone [24].

The bronchial involvement in UC is presented in different forms. The common feature of all forms is basically an inflammation around the epithelial layer of the tracheobronchial system. Most of the reports show changes in the larger bronchi, generation 2–6 and bronchiectasis and also involvement of the trachea [2], [5], [9], [15], [19], [25], [26], [27], [28], [29], [30]. The tracheal involvement can lead to tracheal stenosis [2], [5], [25], [26], [28], [31], [32], [33]. The smaller bronchi can be altered as well [20], [34], [35]. The functional results of Herrlinger et al. [21] confirm these alterations of the bronchial system. The relations concerning UC and bronchial involvement are yet insufficiently elaborated, probably because in most reports the alterations of the bronchi are not seen as a symptom of UC but rather as a separate disease. 

A very characteristic finding in patients with UC is bronchiectasis, which can be diagnosed clinically by mass sputum expectoration or by X-ray, today mainly by CT [1], [2], [4], [5], [6], [8], [19]. Using HRCT, Mahadeva found 13 out of 14 patients with UC to have bronchiectasis [15]. With these data it is obvious that a particular relation exists between bronchitis and colitis in form of a distinct syndrome. The bronchial involvement can take place before the clinical outbreak of UC, but mostly thereafter, with or without a corresponding clinical activity of UC. Therapeutic interventions as IBD modifying drugs or colectomy have no controlling effect of the respiratory manifestations [9], [32], but it is reported that the bronchial alterations respond to corticosteroids [36], as holds true also in our case. 

## Conclusions

The bronchial changes in UC are often reported as necrotising. It is not known, whether the morphological changes in bronchial and colonic tissue are part of a common process. Today it is too early to answer this question. We would not speculate on common pathogenetic mechanisms, although some authors discuss a common inflammatory vulnerability [24] or pulmonary-intestinal mucosal inflammatory cross-talk [37] involving multiple pathogenetic pathways including microbiomics [4]. The accentuated suppurative character of the bronchitis in many of the patients with UC is striking. Massive mucopurulent secretions and frequently a development of cylindrical bronchiectasis as a result of destruction of bronchial wall may show similarities with the changes in the colonic mucosa (Figure 2 (Fig. 2) and Figure 3 (Fig. 3)). Though changes in the bronchial structure can be easily discovered with lung CT [15], [19], [27] unfortunatly this is only seldom performed in patients with UC. There is no systematic prospective study addressing this clinical problem. 

Both our pilot study and the data from literature encourage to look in more details and greater groups concerning the combination of colonic and bronchial inflammation, as well as the relation to smoking, with the conception of a common “colo-bronchitic” syndrome. We assume that this will promote understanding the pathogenesis of both UC and bronchial inflammation.

## Notes

### Competing interests

The authors declare that they have no competing interests.

## Figures and Tables

**Table 1:**
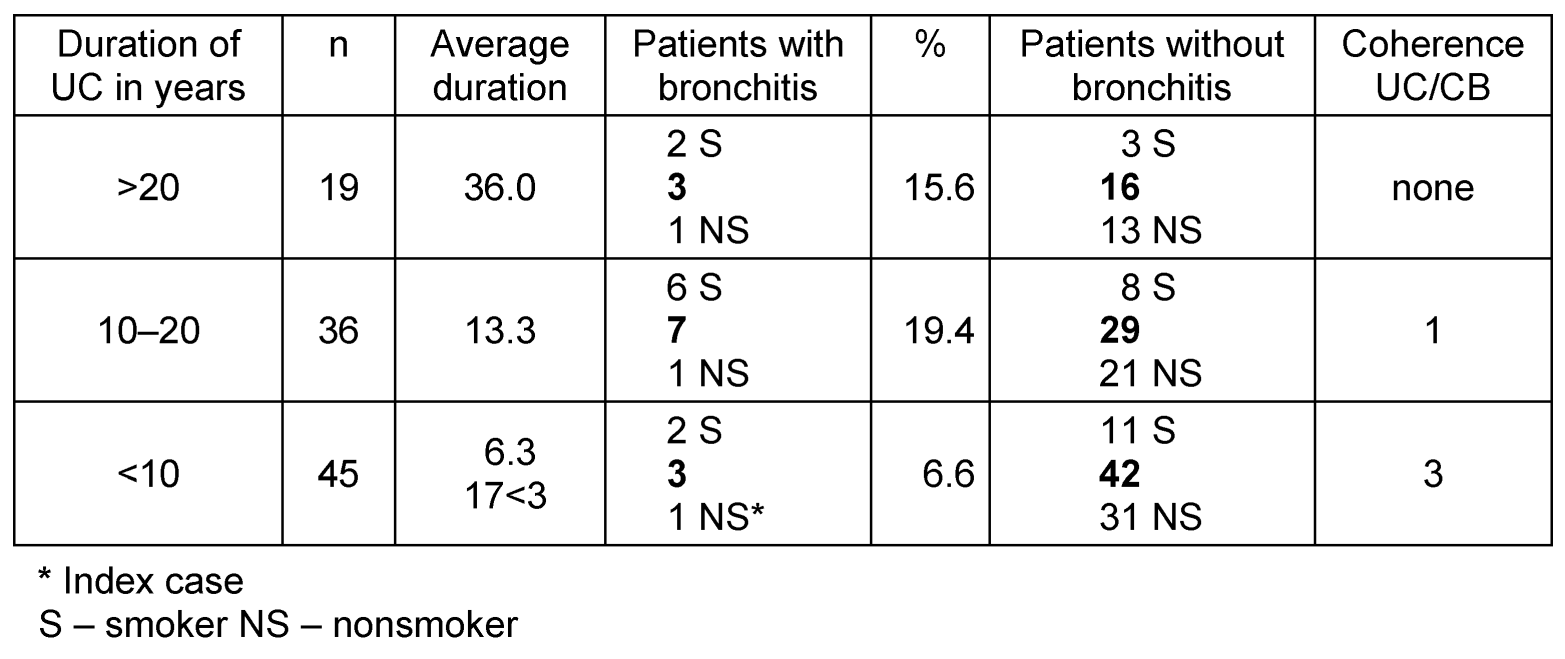
Analysis of 100 patients with ulcerative colitis (UC)

**Table 2:**
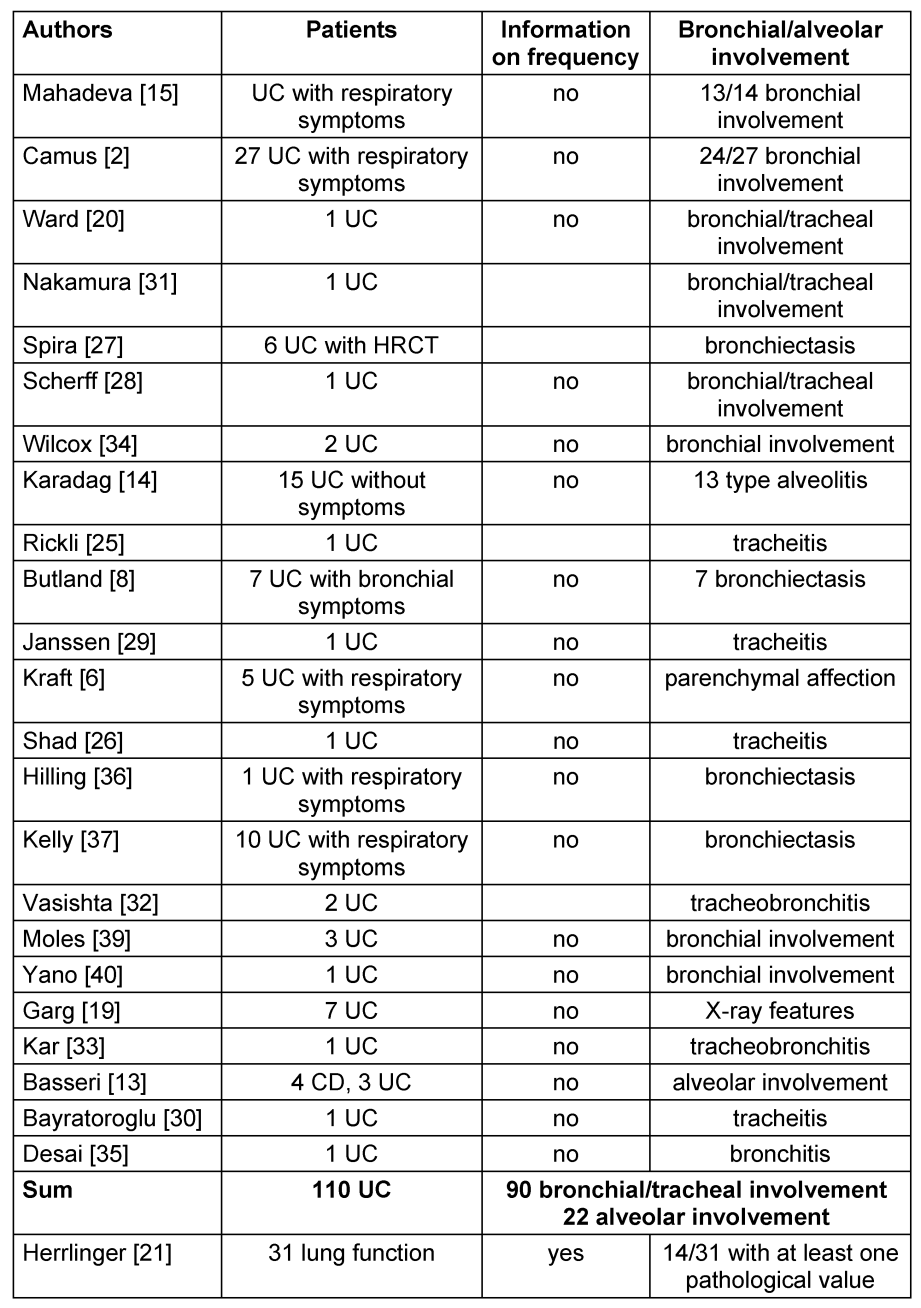
Overview of some previous publications on lung involvement in ulcerative colitis (UC)
